# Supplementary material for: Causal Relationship between Aging and Anorexia Nervosa: A White-Matter-Microstructure-Mediated Mendelian Randomization Analysis
Source: Biomedicines. 2024 Aug 16;12(8):1874. doi: 10.3390/biomedicines12081874 (PMC11351342; doi:10.3390/biomedicines12081874)
Supplement: Supplementary file 1 [file biomedicines-12-01874-s001.zip › Supplementary Figure/Figure S6/Funnel plot of MR analysis for ubm-b-1523 to AN.pdf]

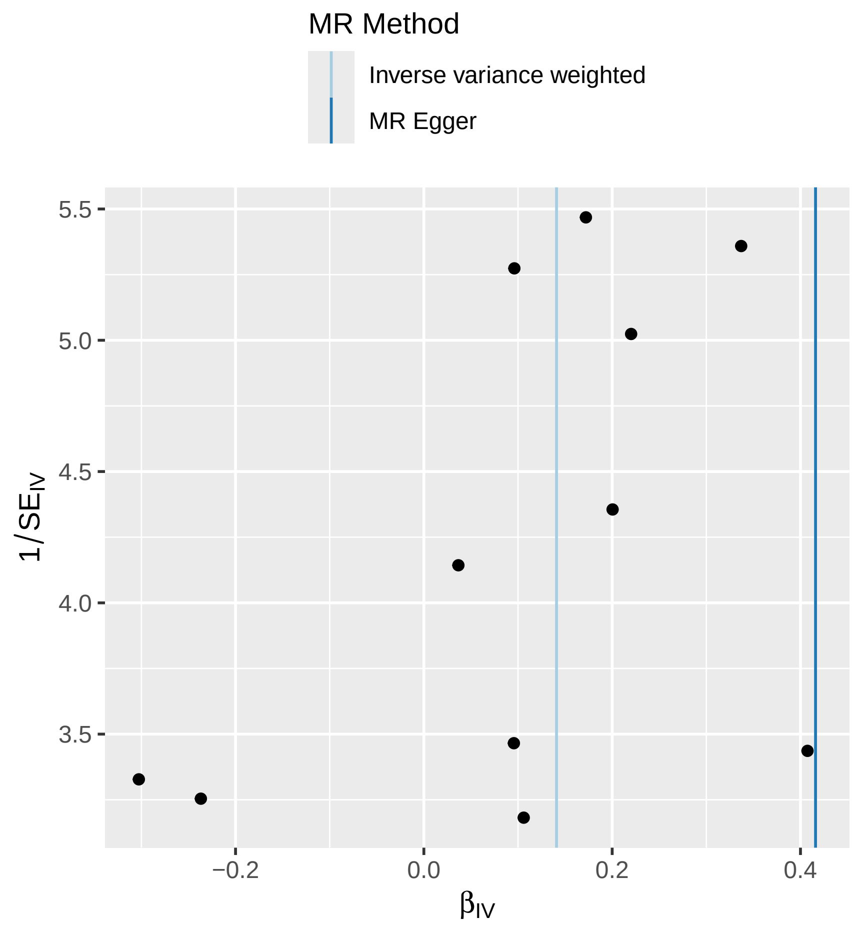

MR Method
Inverse variance weighted
MR Egger
5.5
5.0
4.5
4.0
3.5
−0.2
0.0
0.2
0.4
$1/SE_{IV}$
β_{IV}
